# Ranavirus infections associated with skin lesions in lizards

**DOI:** 10.1186/1297-9716-44-84

**Published:** 2013-09-27

**Authors:** Anke C Stöhr, Silvia Blahak, Kim O Heckers, Jutta Wiechert, Helge Behncke, Karina Mathes, Pascale Günther, Peer Zwart, Inna Ball, Birgit Rüschoff, Rachel E Marschang

**Affiliations:** 1 Fachgebiet für Umwelt- und Tierhygiene, University of Hohenheim, Garbenstr. 30, 70599 Stuttgart, Germany; 2 Chemisches und Veterinäruntersuchungsamt OWL, Westerfeldstr. 1, 32758 Detmold, Germany; 3 Laboklin GmbH & Co. KG, Laboratory for Clinical Diagnostics, Steubenstr. 4, 97688 Bad Kissingen, Germany; 4 Reptilium Terrarien- und Wüstenzoo, Werner Heisenberg Str.1, 76829 Landau, Germany; 5 Import-Export Peter Hoch GmbH, August-Jeanmaire-Str. 12, 79183 Waldkirch, Germany; 6 Klinik für Heimtiere, Reptilien, Zier- und Wildvögel, Stiftung Tierärztliche Hochschule Hannover, Bünteweg 9, 30559 Hannover, Germany; 7 Department of Veterinary Pathology, Faculty of Veterinary Medicine, Utrecht University, Yalelaan 1, P.O. Box 80.158, 3508 Utrecht, The Netherlands; 8 Tierärztliche Gemeinschaftspraxis, Schmarjestraße 52, 22767 Hamburg Altona, Germany; 9 Current address: Laboklin GmbH & Co. KG, Steubenstr. 4, 97688 Bad Kissingen, Germany

## Abstract

Ranaviral disease in amphibians has been studied intensely during the last decade, as associated mass-mortality events are considered to be a global threat to wild animal populations. Several studies have also included other susceptible ectothermic vertebrates (fish and reptiles), but only very few cases of ranavirus infections in lizards have been previously detected. In this study, we focused on clinically suspicious lizards and tested these animals for the presence of ranaviruses. Virological screening of samples from lizards with increased mortality and skin lesions over a course of four years led to the detection of ranaviral infections in seven different groups. Affected species were: brown anoles (*Anolis sagrei*), Asian glass lizards (*Dopasia gracilis*), green anoles (*Anolis carolinensis*), green iguanas (*Iguana iguana*), and a central bearded dragon (*Pogona vitticeps*). Purulent to ulcerative-necrotizing dermatitis and hyperkeratosis were diagnosed in pathological examinations. All animals tested positive for the presence of ranavirus by PCR and a part of the major capsid protein (MCP) gene of each virus was sequenced. Three different ranaviruses were isolated in cell culture. The analyzed portions of the MCP gene from each of the five different viruses detected were distinct from one another and were 98.4-100% identical to the corresponding portion of the frog virus 3 (FV3) genome. This is the first description of ranavirus infections in these five lizard species. The similarity in the pathological lesions observed in these different cases indicates that ranaviral infection may be an important differential diagnosis for skin lesions in lizards.

## Introduction

Ranaviruses (family *Iridoviridae*) are increasingly important pathogens in conservation and medicine of ectothermic vertebrates (fish, amphibians, reptiles). It has been demonstrated that these viruses have caused numerous mass mortality events in various wild and captive amphibian species around the world, reviewed in [1]. Ranaviruses are considered emerging pathogens and are therefore of significant ecological importance [2-4]. In fish, ranaviruses are an important economic factor, as infections have been known to cause severe piscine die-offs in aquaculture farms [5].

Since the late 1990’s, ranaviruses have also been found more frequently in reptiles. Most cases have been described in chelonians on different continents. Affected species include Mediterranean tortoises (Hermann’s tortoises (*Testudo hermanni*), Egyptian tortoises (*Testudo kleinmanni*), marginated tortoises (*Testudo marginata*), and spur-thighed tortoises (*Testudo graeca*)) [6-9], as well as a Russian tortoise (*Testudo horsfieldii*) [10], Burmese star tortoises (*Geochelone platynota*), gopher tortoises (*Gopherus polyphemus*) [11,12], a leopard tortoise (*Geochelone pardalis*) [13], Chinese soft-shelled turtles (*Trionyx sinensis*) [14] and Florida box turtles (*Terrapene carolina bauri*) [12]. In a transmission study, a ranavirus isolated from a Burmese star tortoise caused disease in red-eared sliders (*Trachemys scripta elegans*) and Western ornate box turtles (*Terrapene ornata ornata*) [15]. An increasing number of emerging disease outbreaks attributed to ranavirus infection have been detected in free ranging Eastern box turtles (*Terrapene carolina carolina*) in the United States e.g. [12,16-19].

Infections have been associated with lethargy, anorexia and mortality. Predominant clinical signs in affected animals have been described in the upper respiratory tract (nasal discharge, conjunctivitis, diphtheroid-necrotic stomatitis); in some animals severe subcutaneous cervical edema and “red-neck disease” have been found. Hepatitis, enteritis and pneumonia have been diagnosed in pathological examinations.

Only one case of ranavirus infection in snakes has been documented in Australia [20]. These green pythons (*Morelia viridis*, formerly *Chondropython viridis*) showed ulceration of the nasal mucosa, hepatic necrosis and severe necrotizing inflammation of the pharyngeal submucosa.

Three cases of ranaviral infection in lizards have been published so far: A leaf-tailed gecko (*Uroplatus fimbriatus*) from Germany, which died with ulcerative-necrotizing glossitis and focal necrosis in the liver tested positive for the presence of ranavirus [21]. Another ranavirus was isolated from a wild caught Iberian mountain lizard (*Lacerta monticola*) from Portugal. The animal was also infected with erythrocytic necrosis virus, but no overt disease was documented [22]. Recently, a ranavirus was detected in association with a mass-mortality event in a group of green striped tree dragons (*Japalura splendida*), which were imported into Germany [23]. On pathological examination, granulomatous and necrotizing inflammation of the skin, tubulonephrosis, hyperemia and liver necrosis were found in those animals. An adenovirus (AdV) and invertebrate iridovirus (IIV) were also found in the same group.

In this publication, we describe the first detection of ranaviruses in five additional lizard species. All viruses were distinct from one another and skin lesions were found in association with ranaviral infection in each case.

## Materials and methods

### Case reports

#### Brown anoles (*Anolis sagrei*)

In February 2008, 224 male brown anoles were imported from Florida into southern Germany. On arrival, most animals were dehydrated and a total of 13 animals were found dead. During the next weeks, animals were lethargic and skin lesions (multiple grayish skin alterations up to 3 mm in diameter) were detected. Parasitological investigations at the wholesaler revealed infection with flagellates, cosmocercoids (*Atractis* spp.), trematodes (*Mesocoelium* spp.) and *Coccidia* spp. (*Eimeria* cf. *anolidis*). The infestation was between low to high intensity. Animals were treated with ronidazol (200 mg/L water (Ridzol® 10%, Dr. Hesse Tierpharma GmbH & Co KG, Hohenlockstedt, Germany)) in spray water for five days. Afterwards, antibiotic treatment with enrofloxacine (150 mg/L drinking water (Baytril® 10% oral solution, Bayer AG - Division Animal Health, Leverkusen, Germany)) was administered over a period of five days. Nevertheless, 30% of the group died within two weeks. One animal was submitted for necropsy (AS-1). Pathohistological examination showed a necrotizing dermatitis. Microbiological investigations of liver and kidney revealed a high amount of fungi and a low amount of *Staphylococcus* spp. and *Streptococcus* spp. in the liver. Tissue samples (lungs and affected parts of the skin) from this animal were submitted for virological testing.

A few months later, in May 2008, a group of approximately 50 brown anoles, which were imported by another wholesaler in northern Germany, also developed disease. All showed multiple grayish skin alterations and were in bad condition, an unknown number died, the remaining ones were euthanized. One of the euthanized animals was submitted for pathological examination (AS-2). Histological investigation of the skin lesions, which covered the whole body, showed an ulcerative-necrotizing dermatitis. Organ tissues were pathologically non remarkable. No inclusion bodies were detected in any organ. Samples from the skin were submitted for virological examination.

In February 2011, 97 male and 193 female brown anoles from an allochthonous population near Miami (Florida) were imported into southern Germany by the same wholesaler as in February 2008. 15 animals were dead on arrival. The anoles were kept in terrariums in small groups divided by sex at temperatures ranging between 26 and 27 °C, 30 °C at local sunning spots during the day. At night, the temperature decreased to 22–23 °C. The relative humidity varied between 85-95%. Coprological examination revealed infections with different endoparasites (flagellates, cosmocercoids, trematodes, coccidia) as described in the animals that had been imported in 2008. An increasing number of anoles became lethargic, dehydrated, and maculae were detected on the skin of some animals. Over a period of five weeks, 53% of the imported group died (66% male, 46% female). No pathological changes were detected during a short pathological examination. Antibiotic and antiparasitic treatment with enrofloxacine (150 mg/L drinking water (Baytril® 10% oral solution, Bayer AG - Division Animal Health)) and ronidazol (200 mg/L drinking water (Ridzol® 10%, Dr. Hesse Tierpharma GmbH & Co KG)) over five days did not improve the course of disease, but changes in housing conditions (animals were moved to gauze terrariums in a green house with similar temperatures during the day and direct exposure to the sun, but lowered to 11 °C at night) reduced the mortality rate considerably. One dead brown anole with skin lesions was submitted for virological examination (AS-3).

#### Asian glass lizards *(Dopasia gracilis)*

**Figure 1:**
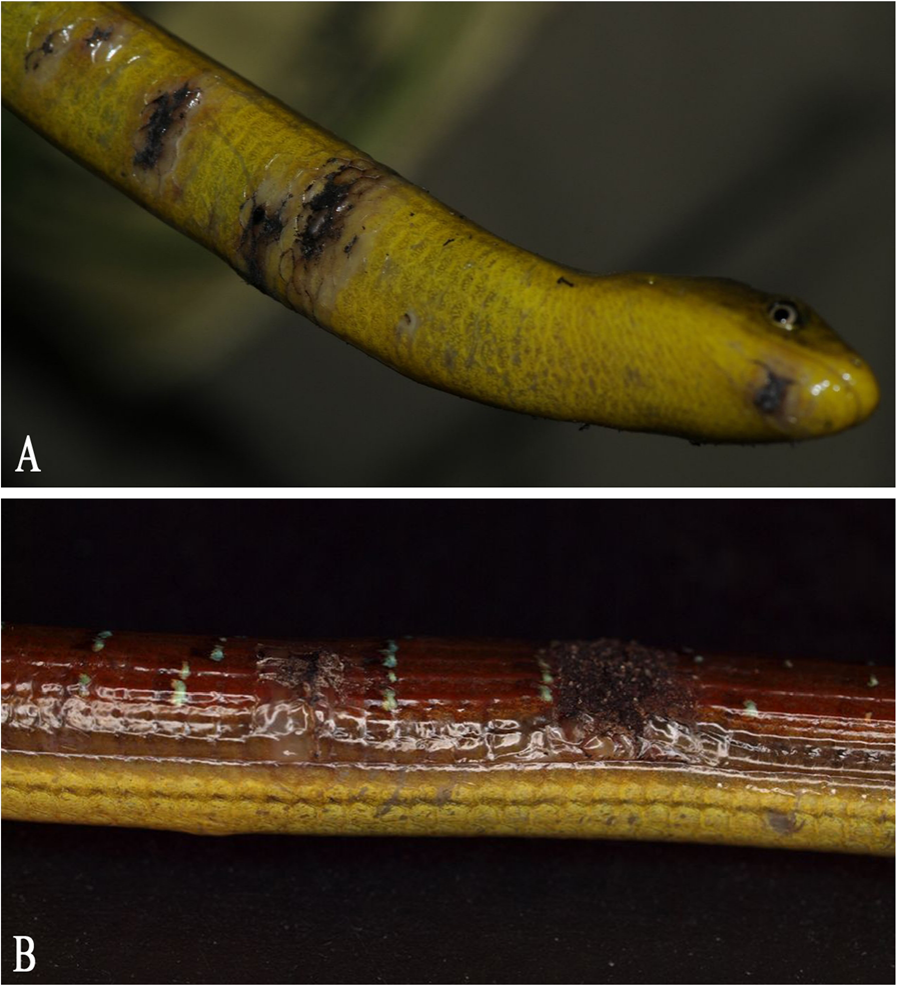
**Ranavirus infected Asian glass lizard (*Dopasia gracilis*). (A)**: skin lesions on the ventral surface of the body. **(B)**: brown crusted skin lesions on the dorsum.

**Figure 2:**
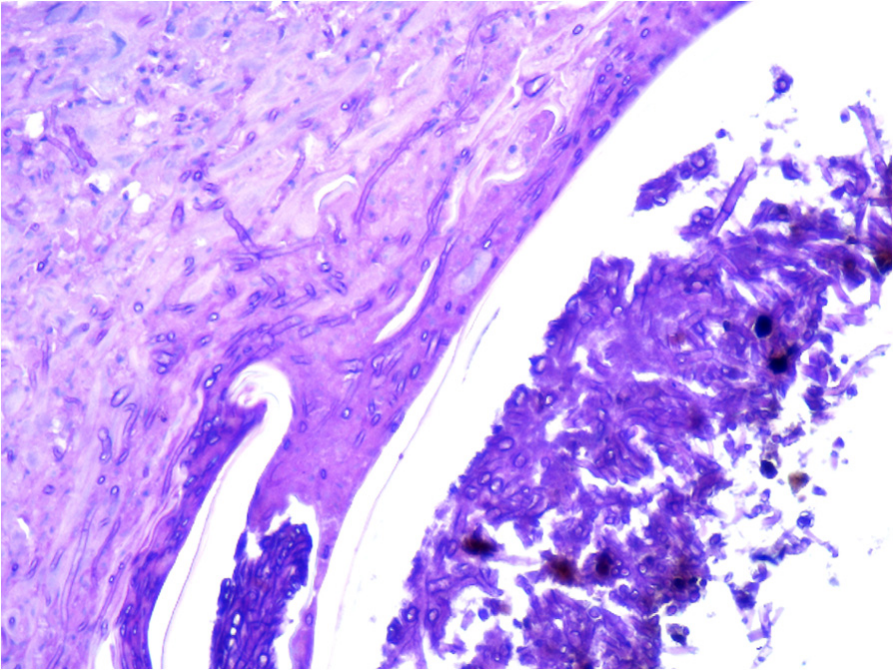
**Histopathological skin lesion (ulcerative dermatitis) of a ranavirus infected Asian glass lizard (*Dopasia gracilis*).** Note the intralesional fungal hyphae invading the dermis. 400 × PAS stain.

In December 2011, 570 illegally imported animals from Asia were confiscated in Germany. Different invertebrates, anurans (horned frogs (*Ceratophrys* spp.)), urodeles (e.g. Chinese fire-bellied newt (*Cynops orientalis*), blue-tailed fire-bellied newts (*C. cyanurus*)), snakes, tortoises (Indochinese box turtles (*Cuora galbinifrons*), big-headed turtles (*Platysternon megacephalon)*, Fly river turtles (*Carettochelys insculpa*), Vietnamese leaf turtles (*Geoemyda spengleri*), Burmese star tortoises (*Geochelone platynota*), Japanese pond turtles (*Mauremys japonica*)), and lizards (e.g. Asian glass lizards (*Dopasia gracilis*), Szechwan japalures (*Japalura* cf. *flaviceps*) and Chinese water skinks (*Tropidophorus* cf. *sinicus*)) were found [24]. A total of 82 Asian glass lizards were divided up and sent to different zoological organizations. Some weeks later, animals in one zoo showed multiple brown-crusted skin lesions (Figure 1A and B) and all of them (25 animals) died during the next weeks. The history of the other confiscated animals is unknown. One Asian glass lizard was submitted for necropsy. The animal was in poor body condition, pathological changes were only detected in the skin. Histologically, a hyperplastic, ulcerative dermatitis was diagnosed. Fungal hyphae invading the dermis were detected within the lesions (Figure 2), no inclusion bodies were found. Skin and mixed organ samples (lungs, liver, kidney, intestine) from this animal were also submitted for virological examination.

#### Green anoles *(Anolis carolinensis)*

**Figure 3:**
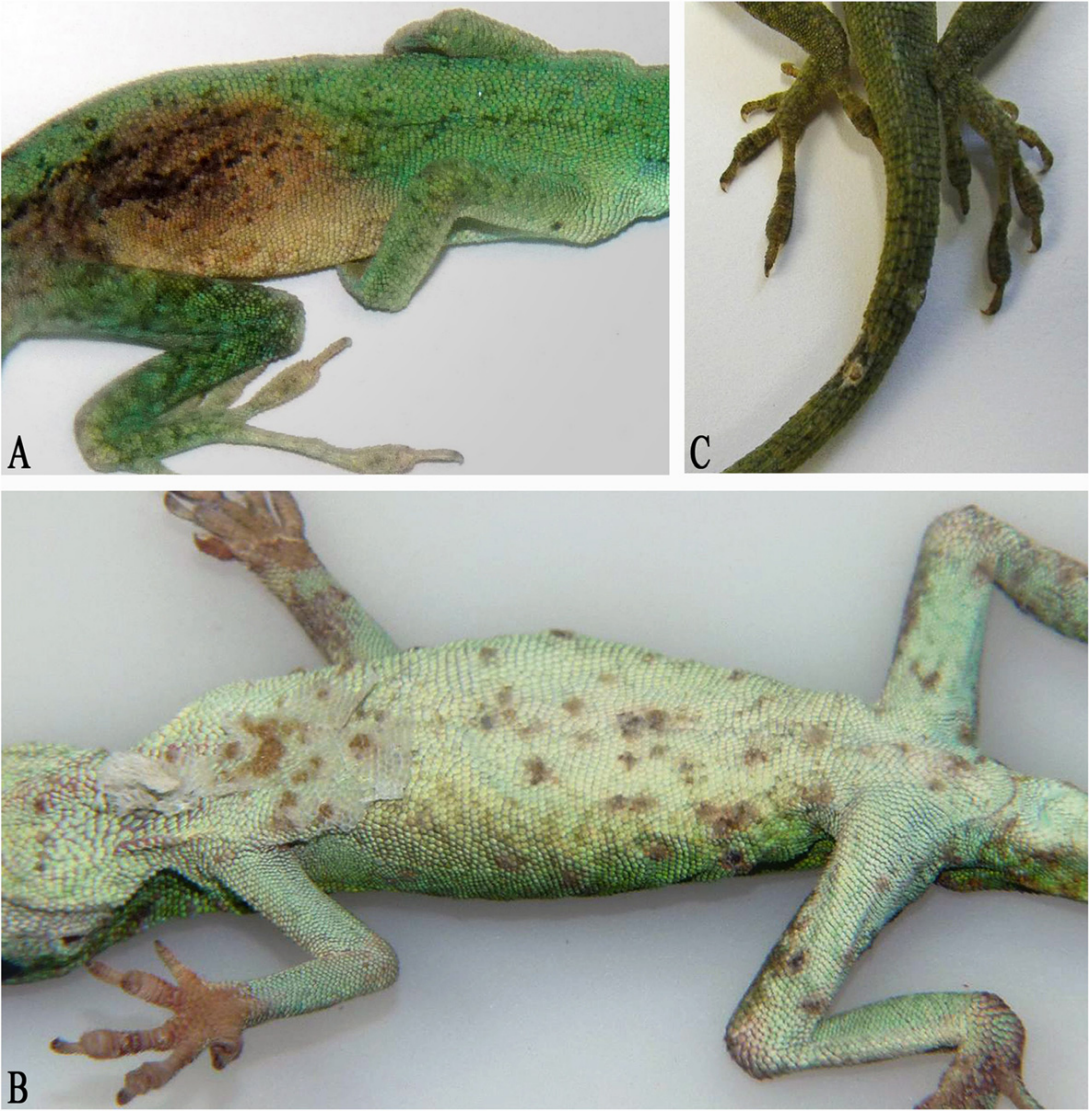
**Skin alterations observed in ranavirus infected green anoles (*Anolis carolinensis*). (A)**: beige gray discoloration of the skin at the lateral abdomen. **(B)**: multiple ulcera on the ventral abdominal surface. **(C)**: grayish lesions on the skin of the tail.

Green anoles (*Anolis carolinensis*) from an autochthonous population in Miami (Florida) were repeatedly imported by a wholesaler to southern Germany. A permanent stock of approximately 300–400 male and 150–200 female animals were housed in small groups (25–30 animals) divided by sex in gauze terrariums in a green house. In summer, the temperature ranged between 25–30 °C during the day and 20–25 °C during the night. In winter, the temperature varied from 10–25 °C during the day to 8–10 °C at night. Between October 2011 and March 2012, a total of 2400 animals (from five deliveries) were obtained by the same wholesaler who imported the brown anoles. Increased mortality rates were observed following these imports (October 2011: 0.8%; November 2011: 0.4%; December 2011: 8%; January 2012: 15%; February 2012: 13%; March 2012: 4%; April 2012: 2%). Parasitological investigations at the wholesaler’s premises revealed a high load of flagellates, a low number of *Coccidia* spp., nematodes (*Oxyuris* spp.), and occasionally tapeworms (*Oochoristica* cf. *anolis*). Short gross pathological examination demonstrated catarrhal enteritis in individual animals. Several animals, which were in poor body condition, were separated from the group. Skin alterations (gray beige areas in the level of the dermis (Figure 3A) and ulcerative dermatitis (Figure 3B)) were detected in some animals. Antiparasitic treatment using ronidazol (200 mg/L drinking water (Ridzol® 10%, Dr. Hesse Tierpharma GmbH & Co KG) over six days) and antibiotic treatment with enrofloxacine (150 mg/L drinking water (Baytril® 10% oral solution, Bayer AG - Division Animal Health) over five days) did not show any positive impact on the diseased animals. One deceased animal with grayish skin lesions (Figure 3C) was submitted for virological examination in April 2012.

#### Green iguanas *(Iguana iguana)*

Adult male and female iguanas from different origins were collected to a single group in a private zoo for a display. Over a period of five years, individual animals developed repeatedly hyperkeratotic skin lesions interrupted by asymptomatic periods. Further examination revealed bacterial infection, and in some animals also dermatomycosis. Depending on the results of microbiological examination, the affected animals were treated locally with an antiseptic (e.g. povidone-iodine solution (Braunol® solution 7.5%, B. Braun Vet Care GmbH, Tuttlingen, Germany)) or with systemic antibiotic and antimycotic therapy, respectively. Neither treatment cured the affected animals. Some iguanas died during the course of disease, some were euthanized, and others did not show clinical signs of disease for years. In May 2012, the last iguana of the group developed disease and a skin biopsy from an affected region was submitted for virological testing. Some weeks later, the animal was euthanized due to the clinical progression of disease.

#### Central bearded dragon (*Pogona vitticeps*)

**Figure 4:**
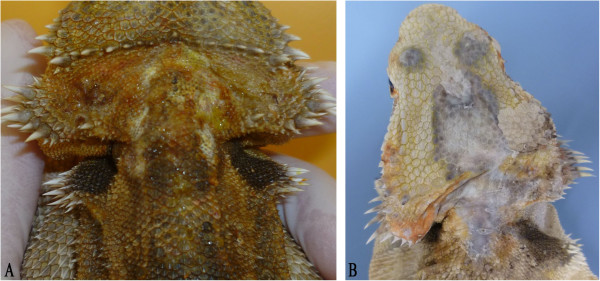
**Skin lesions in a ranavirus infected central bearded dragon (*Pogona vitticeps*). (A)**: pustules on the skin in the region of the neck and the head (August 2011); **(B)**: skin alterations on the head and neck (same animal as Figure 4A in October 2012).

A five-year old male central bearded dragon was presented for medical examination in July 2011 due to the sudden appearance of pustules on the skin in the region of the neck and the head (Figure 4A). In histological examination of a skin biopsy, a purulent dermatitis was diagnosed. A low amount of *Micrococcus* spp. was found. Antimicrobial therapy (trimethoprim/sulfadoxinum, 30 mg/kg (Borgal® 24%, Virbac Tierarzneimittel GmbH, Bad Oldesloe, Germany) administered orally over a period of 21 days) combined with local antiseptic treatment (povidone-iodine solution (Braunol® solution 7.5%, B. Braun Vet Care GmbH) and ethacridine lactate solution (Acridin powder®, Wirtschaftsgenossenschaft Deutscher Tierärzte eG (WDT), Garbsen, Germany)) showed positive effects on the wounds.

**Figure 5:**
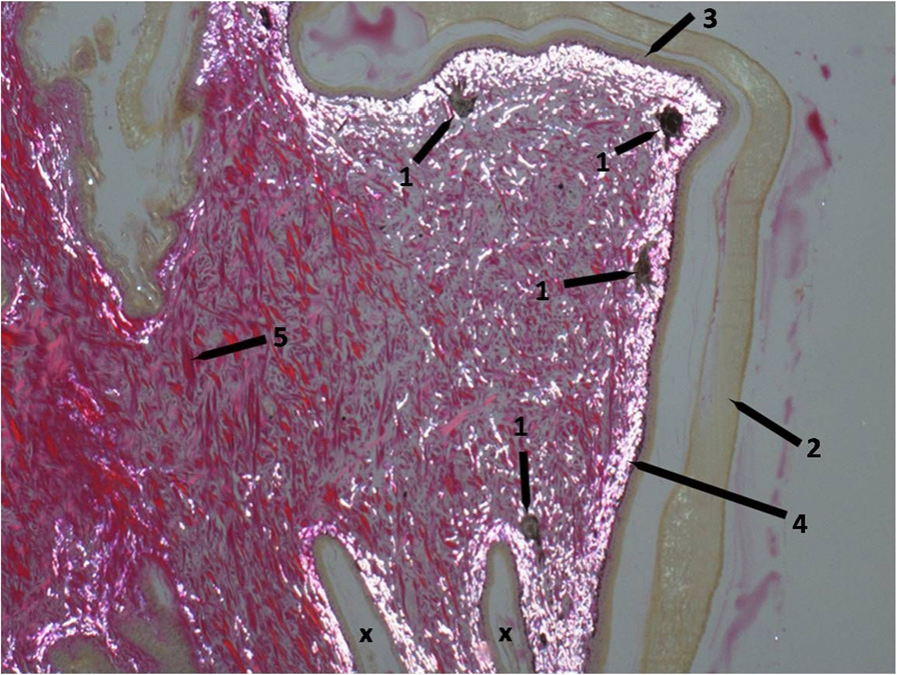
**Histopathological skin proliferation of a ranavirus infected central bearded dragon (*Pogona vitticeps*).** Note the irregularly distributed iridophores and the large melanocytes. 400 × Van Gieson stain; polarized light. 1: melanocytes, 2: horny layer, 3: epidermis, 4: iridophores (white iridescence), 5: connective tissue fibres (red), x: indentations of the normal skin, the right one is bordering the pathological areas.

In October 2012, the animal was presented again. Severe inflammation and necrosis were diagnosed in one leg. Dark skin lesions were detected on the head, neck and back (Figure 4B). Radiographic examination showed a profound lysis of the bones of the hind-foot and the proximal lower leg. Since the owner refused amputation of the diseased leg, the bearded dragon was euthanized. Pathologically, an ichorous myositis with involvement of the bones was diagnosed. The liver was enlarged and pale. Histologically, the epidermis around the skin lesions appeared normal. However, areas of proliferated connective tissue were noted in the cutis and subcutis. Iridophores were irregularly distributed throughout these areas. Individual extremely large melanocytes were detected underneath these areas (Figure 5). No inclusion bodies were detected. Swabs (oral/cloacal) and different organ samples (skin, muscle, heart, intestine, liver, kidney, lungs) were submitted for virological testing.

### Virological examination

Samples submitted for virological testing (Table 1) were placed in 3 mL Dulbecco’s modified Eagles medium (DMEM) (Biochrom AG, Berlin, Germany) supplemented with antibiotics. The samples were sonified, clarified by centrifugation at low speed (2000 × *g*, 10 min), and 200 μL of the supernatant was inoculated onto iguana heart cells (IgH-2, ATCC: CCL-108) in 30 mm diameter Cellstar® tissue culture dishes (Greiner Bio-One GmbH, Frickenhausen, Germany). After incubation (2 h at 28 °C), 2 mL DMEM supplemented with 2% foetal calf serum (FCS) (Biochrom AG), 1% non-essential amino acids (Biochrom AG) and antibiotics were added to each dish. For the samples from the brown anoles, supernatants were filtered using a 0.2 μm filter (Whatman International LTD, Kent, UK) and inoculated onto Russells viper heart cells (VH-2, ATCC: CCL-140) instead of IgH-2.

**Table 1:** Results of virological testing of lizards for the presence of ranaviruses together with additional viruses

**Samples**	**Ranavirus PCR from original samples**	**Virus isolation**	**Ranavirus PCR from virus isolates**	**Additional viruses detected**
**1: *Anolis sagrei***				
AS-1:				
skin, lungs	n.d.	+	+*	
AS-2:				
skin	n.d.	+	+*	
AS-3:				
skin	n.d.	+	+*	Reovirus (isolated)
pooled organs	n.d.	+	+*	
**2: *Dopasia gracilis***				
skin	+	+	+*	IIV (isolated)
mixed organs	+	-		
**3: *Anolis carolinensis***				
skin	+	+	+*	IIV (PCR)
liver	+	+	+	
small intestine	+	+	+	AdV (PCR)
**4: *Iguana iguana***				
skin	+*	-		
**5: *Pogona vitticeps***				
skin	+*	-		
muscle	+	-		
heart	+	-		
oral/cloacal swab	-	-		AdV (PCR)
intestine, liver kidney, lungs	-	-		

+: positive; -: negative; n.d.: not done; *:PCR product sequenced; IIV: invertebrate iridovirus; AdV: adenovirus.

Tissue cultures were observed twice a week for cytopathic effects (CPE); cultures showing no CPE after 2 weeks of incubation were frozen, thawed and reinoculated onto the respective cell line for a second passage.

DNA was extracted from the supernatant of the original sample or cell culture supernatant of the isolates using a DNeasy Kit (Qiagen, Hilden, Germany) and a polymerase chain reaction (PCR) for the detection of ranaviruses was performed [8,10] using the sense primer Ol T1 (5’-GACTTGGCCACTTATGAC-3’) and the antisense primer Ol T2R (5’-GTCTCTGGAGAAGAAGAAT-3’), targeting a 500 bp portion of the major capsid protein (MCP) gene. PCR’s for the detection of adenoviruses (AdVs) [25] and invertebrate iridovirus (IIV) [26] were also performed during routine virological examination of lizard samples in our laboratory. If a CPE consisting of syncytical giant cells appeared, a PCR [27] was done additionally to confirm the presence of a reovirus.

PCR products from each positive animal were agarose gel purified (peqGOLD gel extraction kit, Peqlab Biotechnologie GmbH, Erlangen, Germany) and submitted to MWG Biotech GmbH (Ebersberg, Germany) for sequencing from both directions. The sequences were edited and compared using the STADEN Package version 2003.0 Pregap4 and Gap4 programmes [28]. The sequences were then compared to those in GenBank (National Center for Biotechnology Information, Bethesda, Maryland, USA) online [29] and to the local ranavirus database of the Fachgebiet für Umwelt und Tierhygiene at the University of Hohenheim.

## Results

Results of virological testing are listed in Table 1. Ranaviruses were detected by PCR in different samples (skin, organs) of each species described, but virus isolation methods were only successful for the brown and green anoles and the Asian glass lizard. In addition to the ranaviruses, a reovirus was isolated from the skin of a brown anole (animal from 2011) and IIV was isolated from the skin of the Asian glass lizard. By PCR, IIV was also detected in the skin of the green anole; AdV was detected in the intestine from the same animal and in the swabs from the central bearded dragon.

Sequencing of the obtained 500 bp portion of the MCP gene demonstrated that the three isolates from the brown anoles (both from 2008 and the isolate from 2011) were identical to one another, but that each of the ranaviruses detected in the five different lizard species were distinct from one another (Table 2). The sequence from the ranavirus of the green anole (*Anolis carolinensis* ranavirus, ACRV) was 100% identical to the corresponding nucleotide (nt) sequences from frog virus 3 (FV3) [GenBank:AY548484], the type species of the genus ranavirus. The sequence from the isolates from the brown anoles (*Anolis sagrei* ranavirus, ASRV) also showed the highest identity values (99.6%) to the corresponding nucleotides of FV3. Comparing amino acid (aa) sequences, both anole ranaviruses were 100% identical to FV3. The ranavirus detected in the green iguana (*Iguana iguana* ranavirus, IIRV) showed 100% identity to corresponding nucleotide sequences from fish ranaviruses (European sheatfish virus (ESV) [GenBank:JQ724856] and European catfish virus (ECV) [GenBank:FJ358608]). Obtained nucleotide sequences from the central bearded dragon (*Pogona vitticeps* ranavirus, PVRV) were 100% identical to sequences from ranaviruses detected in different tortoises from Germany (= isolate from *Testudo marginata* (CU60/09), *Testudo hermanni* (5187/07) and *Testudo kleinmanni* (882/96) [9]). Partial sequence from the Asian glass lizard (*Dopasia gracilis* ranavirus, DGRV) was most closely related (99% nt identity, 100% aa) to *Rana tigrina* ranavirus [GenBank:AY033630].

**Table 2:** Ranavirus sequence identity of the analyzed partial sequences of the MCP gene (502 nt) in %

	**ASRV**	**DGRV**	**ACRV**	**IIRV**	**PVRV**	**FV3**	**SSTIV**	**CH8/96**	**ESV/ECV**	**RTV**	**TRV**
ASRV		98.0	**99.6**	96.4	98.0	**99.6**	99.4	98.2	96.4	98.2	98
DGRV	98.8		98.4	96.0	97.6	98.4	98.6	97.8	96	**99.0**	97.6
ACRV	**100**	98.8		96.8	98.4	**100**	99.8	98.6	96.8	98.6	98.4
IIRV	95.8	96.4	95.8		96.8	96.8	97.0	98.2	**100**	96.2	96.8
PVRV	98.2	98.2	98.2	95.2		98.4	98.6	97.8	96.8	97.8	**100**
FV3	**100**	98.8	**100**	95.8	98.2		99.8	98.6	96.8	98.6	98.4
SSTIV	99.4	99.4	99.4	96.4	98.8	99.4		98.8	97.0	98.8	98.6
CH8/96	97.6	97.6	97.6	98.2	97.0	97.6	98.2		98.2	98. 0	97.8
ESV/ECV	95.8	96.4	95.8	**100**	95.2	95.8	96.4	98.2		96.2	96.8
RTV	98.8	**100**	98.8	96.4	98.2	98.8	99.4	97.6	96.4		97.8
TRV	98.2	98.2	98.2	95.2	**100**	98.2	98.8	97.0	95.2	98.2	

The five different lizard ranaviruses detected in this study are presented in comparison to other ranaviruses from amphians, reptiles and fish. The upper diagonal shows the values for the nucleotide sequence identity, the amino acid identity values are provided in the lower diagonal. Closest identities are highlighted in bold.

ASRV = *Anolis sagrei* ranavirus, DGRV = *Dopasia gracilis* ranavirus, ACRV = *Anolis carolinensis* ranavirus, IIRV = *Iguana iguana* ranavirus, PVRV = *Pogona vitticeps* ranavirus, FV3 = Frog virus 3 [GenBank:AY548484], SSTIV = Soft-shelled turtle iridovirus [GenBank:EU627010], CH8/96 = *Testudo hermanni* ranavirus [GenBank:AF114154], ESV = European sheatfish virus [GenBank:JQ724856], ECV = European catfish virus [GenBank:FJ358608], RTV = Rana tigrina ranavirus [GenBank:AY033630], TRV = Tortoise ranaviruses (= isolates from *Testudo marginata* (CU60/09), *Testudo hermanni* (5187/07) and *Testudo kleinmanni* (882/96) [9]).

## Discussion

Five different ranaviruses were detected in five different lizard species (*Dopasia gracilis, Anolis sagrei, Anolis carolinensis, Iguana iguana, Pogona vitticeps*). Skin lesions such as purulent to ulcerative-necrotizing dermatitis and hyperkeratosis were found in association with ranaviral infection in all cases.

Various infectious and non-infectious agents can cause skin lesions in lizards. In captive reptiles, deficiencies in husbandry (e.g. burns from heat sources, unhygienic conditions with excessive humidity and moisture, too cold environment temperatures, exposure to toxic substances, inappropriate nutrition or overcrowding resulting in injuries from other animals) can provoke skin diseases or are often a predisposing factor. Different parasites (e.g. acariasis, ticks, larval ascarids) or neoplasms (e.g. fibrosarcoma, melanoma) can also affect the skin. Secondary infections with bacteria (mostly gram-negative, e.g. *Aeromonas* spp., *Pseudomonas* spp., *Klebsiella* spp.) or dermatomycosis are common findings in microbiological examinations. Some primary pathogens have also been described: *Chrysosporium anamorph of Nannizziopsis vriesii* (CANV) is the etiologic agent of an emerging deep fungal dermatitis (“yellow fungus disease”) in bearded dragons [30]; *Devrisea agamarum* is frequently isolated from spiny tailed lizards (*Uromastyx* spp.) with dermatitis and cheylitis [31].

A number of viruses have been detected in connection with skin alterations in reptiles: fibropapillomatosis in sea turtles and grey patch disease in green turtle hatchlings (*Chelonia mydas*) seem to be caused by herpesviruses; a distinct herpesvirus has also been detected in papillomas of green lizards (*Lacerta viridis*). Poxviruses have been found repeatedly in association with skin lesion in crocodilians and pox-like viruses have also been detected sporadically in other reptiles, reviewed in [32]. Three different viruses have been found in a European green lizard (*Lacerta viridis*) with wart-like skin lesions: herpes-like, reo-like and a papilloma-like virus [33]. Papillomaviruses have also been found in different turtles with small white papules [34,35]. Several lizard species showing skin lesions (a frilled lizard (*Chlamydosaurus kingii*) with pox-like skin lesions, as well as a green iguana (*Iguana iguana*) and a spiny tailed lizard (*Uromastyx* spp.) with hyperkeratosis) [36,37] have been tested positive for the presence of IIV (family *Iridoviridae*)*.*

In European amphibians, acute ranavirus infection has been described in association with systemic haemorrhages, whereas chronic ranaviral disease has been characterized by skin ulcerations without gross internal lesions, reviewed in [38]. Skin ulceration in association with ranaviral disease has also been described in fish, reviewed in [39]. In addition to other clinical signs, cutaneous abscessation has been described in a group of captive Eastern box turtles with ranavirus infection [19] and necrotizing inflammation of the skin was also diagnosed in a group of green striped tree dragons with multiple viral infections, including ranavirus [23]. Since ranaviral infection causes skin alterations in other susceptible species and ranaviruses were detected in every studied lizard species in the skin (Table 1), we hypothesize that the skin lesions observed in these animals were caused by ranaviruses. Infections with detected bacteria and fungi were most likely secondary. This is supported by the fact that neither antibiotic nor antimycotic treatment showed any positive effect (at least in the long run).

It has been demonstrated previously that some ranaviruses are able to switch hosts between different chelonian families [6,15] and that an amphibian ranavirus (Bohle iridovirus) can even cause disease in turtles [40]. Based on the 500 bp portion of MCP gene sequenced in this study (Table 2), three of the detected ranaviruses from lizards (ASRV, ACRV, DGRV) were most closely related to amphibian ranaviruses (FV3, RTV). One ranavirus (IIRV) was identical to ranaviruses from fish (ECV/ESV) and only one ranavirus (PVRV) showed 100% identity to another reptilian ranavirus detected in European tortoises [6,9]. These findings correspond with previous analyses of the full genome from another reptilian ranavirus (soft-shelled turtle iridovirus) suggesting that this virus was transmitted from amphibians to reptiles [41]. However, since the MCP gene is a highly conserved gene and only partial sequences have been studied, phylogenetic analysis is limited and the origin of the detected ranaviruses in these cases remains unclear. More sequencing work should be done to understand the relationships of these viruses.

Since all three identical ranaviruses from brown anoles (ASRV) detected in the years 2008 and 2011, as well as the slightly different ranavirus from a green anole (ACRV) clustered most closely to another American ranavirus (FV3), we speculate that these anoles brought the virus with them into Germany from the United States. A weakened immune system due to the stress of transport, dehydration and coinfections with endoparasites - as well as with additional viruses in some animals (reovirus, AdV and/or IIV) - may have favoured the clinical development of disease. By characterizing an MHC class I locus in different populations of common frogs (*Rana temporia*) in the UK infected by a ranavirus, it has been hypothesized that the animals are adapting to the presence of ranavirus [42]. It would be interesting to test the populations of anoles in Florida for the presence of ranavirus, to find out if they are subclinically infected.

At least one lizard species (Asian glass lizard) had been in contact with other reptilian and amphibian species prior to testing. Since ranaviruses are able to switch between hosts, it is possible that other confiscated species from this case were also infected. Unfortunately, no data about these animals is available.

During the last decade, the reports of ranavirus infections in reptiles have markedly accelerated. The rising awareness of these viruses in chelonians as important infectious agents causing ulcerative-necrotizing stomatitis may have contributed to the high number of case reports in these species. The frequent detection of ranaviruses in lizards during this study could be related to the use of more sensitive diagnostic methods (PCR). In addition it may be surmised that the global trade of reptiles and amphibians in combination with the wide host range of ranaviruses has strengthened the emergence of the infection. This is of significant importance for wild and captive animals. Ranaviral disease should therefore be considered as a possible differential diagnosis for dermatitis in different species of lizards and newly imported animals should be quarantined.

## Competing interests

The authors declare that they have no competing interests.

## Authors’ contributions

ACS carried out the virological testing for the presence of ranaviruses (including sequencing) and drafted the manuscript. SB carried out the pathological examination of the *Anolis sagrei* and isolated the viruses from the *Anolis sagrei*. She also wrote the corresponding parts of the paper. KOH carried out the pathological examination of the *Dopasia gracilis* and provided pictures. JW treated the *Dopasia gracilis* and the *Iguana iguana* and provided clinical data and pictures. HB treated the *Anolis sagrei* and the *Anolis carolinensis*, performed coprological examination, short pathological examination and provided clinical data and pictures. KM and PG treated the *Pogona vitticeps* and provided clinical data and pictures of the diseased animal. PZ carried out the pathological examination of the *Pogona vitticeps* and provided the histology picture. IB carried out the virological testing for the presence of AdV. BR treated the *Anolis sagrei* in northern Germany and provided clinical data. REM conceived of the study, participated in its design and coordination, advised ACS during her practical work and helped to draft the manuscript. All authors read and approved the final manuscript.
